# Is There a Role for Vitamin D in Amyotrophic Lateral Sclerosis? A Systematic Review and Meta-Analysis

**DOI:** 10.3389/fneur.2020.00697

**Published:** 2020-07-31

**Authors:** Débora Lanznaster, Theodora Bejan-Angoulvant, Jorge Gandía, Helene Blasco, Philippe Corcia

**Affiliations:** ^1^UMR 1253, iBrain, University of Tours, Inserm, Tours, France; ^2^CHRU Tours, Medical Pharmacology Department, Tours, France; ^3^Université de Tours, Tours, France

**Keywords:** amyotrophic lateral sclerosis, vitamin D, meta-analysis, systematic review, biomarker

## Abstract

**Background:** Amyotrophic lateral sclerosis (ALS) is a neurodegenerative condition characterized by the progressive loss of motor neurons. Patients usually die 3–5 years after diagnosis from respiratory failure. Several studies investigated the role of vitamin D as a biomarker or a therapeutic option for ALS patients. To clarify the scientific evidence, we performed a systematic review and different meta-analyses regarding the potential role of vitamin D in ALS.

**Methods:** We performed a systematic review of clinical trials, cohorts, and case–control studies retrieved from PubMed, EMBASE, and Cochrane databases reporting vitamin D levels as a putative biomarker for ALS diagnosis or prognosis or the effect of vitamin D supplementation in ALS patients. Whenever possible, data were pooled using a random-effects model, with an assessment of heterogeneity.

**Results:** Out of 2,996 articles retrieved, we finally included 13 research articles, 12 observational studies (50% prospective), and 1 clinical trial. We found that ALS patients had slightly lower levels of vitamin D than controls (mean difference −6 ng/ml, 95% CI [−10.8; −1.3]), but important confounding factors were not considered in the studies analyzed. We found no relationship between vitamin D levels and ALS functional rate score—revised (ALSFRS-R), with highly heterogeneous results. Discordant results were reported in three studies regarding survival. Finally, five studies reported the effects of vitamin D supplementation with discordant results. Two of them showed a small improvement, whereas two others showed a deleterious effect on ALSFRS-R. One very small clinical trial with important methodological limitations showed some improvement in ALSFRS-R with high doses of vitamin D compared with normal doses.

**Conclusions:** Our review did not find evidence to support the role of vitamin D on ALS diagnosis, prognosis, or treatment. Most studies had important limitations, mostly regarding the risk of bias for not considering confounding factors. Vitamin D supplementation should be offered to ALS patients to avoid other health issues related to vitamin D deficiency, but there is not enough evidence to support the use of vitamin D as a therapy for ALS.

## Introduction

Vitamin D deficiency has been associated with diverse pathologies, from cardiovascular diseases to cancer. However, an umbrella review performed in 2014 could not draw any conclusion about the relationship between vitamin D levels or vitamin D supplementation and various conditions, such as skeletal, cardiovascular, autoimmune, metabolic diseases, or even cognitive disorders like Alzheimer's disease (1). Interestingly, amyotrophic lateral sclerosis (ALS) was not included in the list of conditions examined by this study. Since then, several studies have been investigating the possible relationship between vitamin D and ALS, its role ranging from biomarker to therapy.

ALS is a neurodegenerative disease characterized by the progressive loss of motor neurons that leads to paralysis and respiratory failure 3–5 years after the onset of symptoms (2). Only two drugs are approved for ALS treatment, with only moderate efficacy on delaying disease progression or increasing survival, despite the large number of new and old drugs evaluated in clinical trials (3, 4). These successive failures could be related to the fact that ALS is diagnosed 1 year after the onset of symptoms, a critical period for initiation of treatments that could prevent motor neuron degeneration. This delay is partly due to the absence of reliable biomarkers for the diagnosis of ALS, even if several candidates have been proposed (5–9). Regarding the role of vitamin D in ALS, studies reported very contradictory results, going from protective (10–14), to detrimental (15, 16).

To determine if there is scientific evidence supporting some role for vitamin D in ALS patients, we performed a systematic review of studies reporting data about any potential relationship between vitamin D and ALS. We aimed to investigate if vitamin D levels could be either used as a biomarker for ALS diagnosis or correlated to disease prognosis or progression and if vitamin D supplementation could improve clinical outcomes in ALS patients.

## Methods

### Search Strategy and Studies Selection

One reviewer (DL) searched PubMed, Embase, and Cochrane databases using the algorithm presented in Table 1, for studies published until September 2019. The search was limited to humans but without other limitations (for age or sex or language, for example). After screening on title, abstract, and types of studies, the full text of eligible studies published in English, Spanish, French, and Portuguese were retrieved for further analysis. Titles and abstracts of the retrieved studies were screened to select those that reported some relation between ALS and vitamin D; then, a second reviewer (JG) checked the titles and abstracts to confirm the selection. We considered studies that reported data about the role of vitamin D levels as a biomarker for ALS diagnosis, for vitamin D levels as a prognostic factor for disease or vitamin D supplementation effect on clinical outcomes. To address these relations, we considered studies that were clinical trials, cohort studies, or case–control studies and were reported in research articles or abstracts from specialized congresses. Although case reports and series of individual case reports were initially considered, we finally decided to exclude them because no solid conclusions could be drawn out of them either way. A full-text article was then examined to further confirm eligibility of studies. Any discrepancies were resolved after discussion with a third reviewer (TBA). The protocol was registered in the International Prospective Register of Systematic Reviews (preregister number: 154572) and is available on-demand to the corresponding author of this study. The Preferred Reporting Items for Systematic Reviews and Meta-Analyses (PRISMA) checklist is included as supplementary material (17) (Supplementary File 2).

**Table 1:** Combination of terms used in our search strategy for the PubMed database that was adapted for the other two databases.

Vitamin D domain	Vitamin d/ or 25-OHD.mp. or 25 hydroxyvitamin D.mp. or cholecalciferol/or colecalciferol.mp. or hydroxycholecalciferols/or hydroxycolecalciferols.mp. or calcifediol/or dihydroxycholecalciferols/ or dihydroxycolecalciferols.mp. or calcitriol/ or 24,25-dihydroxyvitamin d 3/ or 24,25-OH2 D3.mp. or ergocalciferols/ or dihydrotachysterol/ or 25-hydroxyvitamin d 2/ or 25- OHD2.mp. or 1,25-dihydroxyvitamin d.mp. or 1,25-OH2 D.mp. or 1,25- dihydroxyvitamin d2.mp. or 1,25-dihydroxyergocalciferol.mp. or 1,25-OH2 D2.mp. or 1,25-dihydroxyvitamin d3.mp. or 1,25-OH2 D3.mp. or ergocalciferols/ or vitamin D2.mp. or vitamin D 2.mp. or vitamin D3.mp. or vitamin D 3.mp
AND	
ALS domain	Amyotrophic lateral sclerosis or ALS or motor neuron disease

### Quality Assessment (Risk of Bias)

We used different tools to analyze the risk of bias in the retrieved studies in accordance with the type of study analyzed. RoB 2 tool was used to evaluate a randomized clinical trial (RCT) (18), whereas ROBINS-I was used to evaluate the risk of bias in the non-randomized trials (19). For the studies analyzing the prevalence of vitamin D deficiency in ALS patients vs. control subjects, the risk of bias was assessed according to Hoy et al. (20). Finally, we conducted the Quality in Prognosis Studies (QUIPS) analysis to evaluate the risk of bias in studies that evaluated the prognostic role of vitamin D (21). The overall risk of bias was considered low if all domains were at low risk or with only one at a moderate level; high if at least one domain was at high risk or >3 classified as moderate; unknown if no judgment could be made; and moderate otherwise (22). Each domain described for each tool was classified according to the risk of bias by one researcher (DL) and revised by a second researcher (JG). TBA was consulted for a final decision when a disagreement on classifications occurred.

### Data Extraction

For each study, the following data were collected: first author, year of publication, journal of publication, data regarding study design, data about study population characteristics, and data about vitamin D dosage or supplementation. To make a quantitative synthesis, the following data were collected: (1) numerical values for vitamin D levels for ALS patients and controls and any informed statistical index; (2) statistical index of the relationship between vitamin D levels and functional score and its confidence interval (CI) and/or the *p*-value; (3) statistical index of the relationship between vitamin D levels and patient survival (hazard ratio, HR) and its CI and/or the *p*-value; and (4) the statistical index of the correlation between vitamin D levels in patients receiving supplementation and the functional score. Data were extracted by one author (DL) and then checked for accuracy by another author (JG).

### Endpoints and Comparisons

We considered three types of comparisons: (1) vitamin D levels in patients with ALS vs. patients without ALS/healthy controls; (2) the relation between vitamin D levels and either survival of patients or their functional disability as expressed by ALS Functional Rate Score—Revised (ALSFRS-R); (3) survival or ALSFRS in patients with and without vitamin D supplementation. Outcomes considered were ALS diagnosis, survival, and ALSFRS-R.

### Statistical Analysis

For each study, we collected either aggregate, raw data or statistics used to describe the levels of vitamin D in ALS patients compared with those in control subjects or the relationship between vitamin D and prognosis (survival or ALSFRS). Whenever we could have sufficient information (at least three studies reporting similar aggregate data or statistics), we used an inverse variance method to pool the data. We chose to perform a random-effects model, as we anticipated high heterogeneity, as we had already shown in a previous systematic review and meta-analysis (9). We assessed heterogeneity by using Cochrane's Q test and *I*^2^; heterogeneity was considered significant when *p* < 0.10, as major if *I*^2^ > 70%, important if *I*^2^ > 50%, or moderate if *I*^2^ <50%. Publication bias was explored by funnel plot and asymmetry tests if 10 or more studies could be included. Meta-analyses were performed using RStudio (23) package meta (24).

## Results

**Figure 1:**
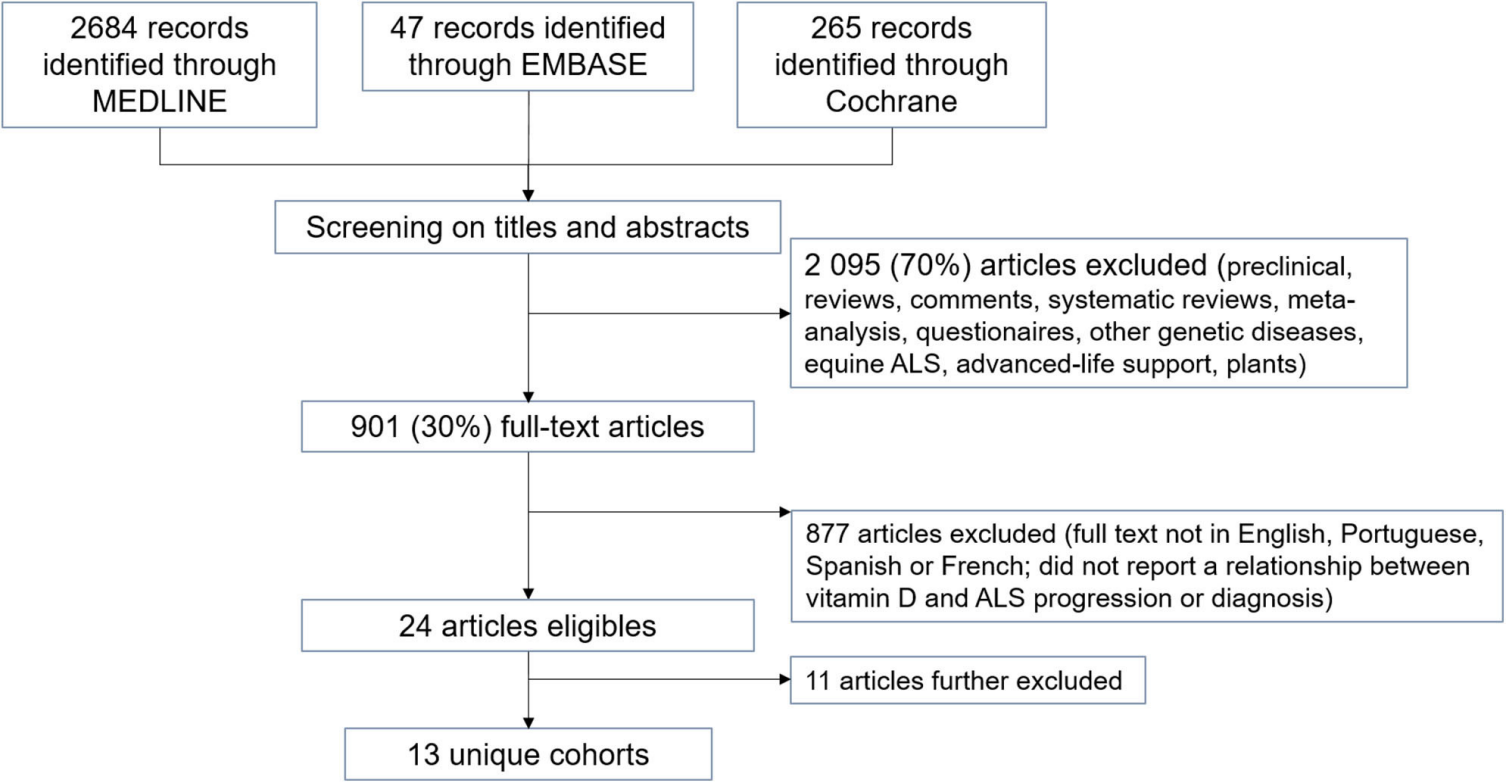
Flow diagram of literature search results for our systematic review.

Our search strategy allowed us to retrieve 2,996 studies. After the screening of titles, abstracts, and types of study, we excluded 2.095 studies (70%). After full-text evaluation of the remaining studies, 24 studies reported some relationship between vitamin D levels or supplementation and ALS diagnosis or progression (Figure 1).

### Articles Further Excluded

After evaluation, seven studies were further excluded from the analysis. Two were case reports: Moling et al. (25) reported a 39-year-old man with ALS and hepatosplenic schistosomiasis, high IL-17 levels, and low vitamin D (14 ng/ml); Shanmugarajah and Archer (26) reported a 69-year-old man bearing the C9orf72 expansion and normal vitamin D levels. Another study reported the mean vitamin D levels of three ALS patients in comparison with other neurological conditions, such as stroke and multiple sclerosis (27). Yanagihara et al. (28) analyzed vitamin D levels in the Chamorros population in the Guam territory. The other two studies evaluated nutritional intake as a source of vitamin D but did not report quantifiable results; moreover, although Gallo et al. (29) said that vitamin D intake was protective against ALS, Dawczynski et al. (30) reported no difference regarding vitamin D and disease progression. One retrieved paper (31) was later retracted (32) (due to “concerns about data integrity and scientific misconduct”) and, therefore, was not included in the analysis.

Besides, four of the retained abstracts and one paper were all published by the group of Camu and co-workers and presented results from the same cohort of ALS patients, so we only included the paper (13) and the more recent abstract (14). Another group published an abstract and a paper based on the same cohort (33), so we only included the paper.

### Summary of Included Studies

Included studies reported (a) the levels of vitamin D in ALS patients compared with those in control subjects (4 studies: 163 ALS and 276 control patients); (b) correlations of levels of vitamin D with clinical markers of prognosis (as ALSFRS-S or survival; 9 studies: 986 ALS patients); or c) effect of supplementation with vitamin D on clinical parameters of progression (3 studies: 77 vitamin D-treated ALS patients, 41 non-treated ALS patients). Of the retained studies, six were retrospective (13, 16, 33–36) and seven prospective (14, 15, 37–41). Four studies were only available as abstracts (14, 34, 35, 38). Twelve were observational studies, and one was described as an RCT (41). No study reported the inclusion (or exclusion) of familial ALS cases, except Cortese et al. (38), who reported the inclusion of only sporadic cases in the evaluated cohort. The characteristics of ALS patients from the retained studies are presented in Table 2. Overall, studies sorted patients according to their levels of vitamin D, and in the majority of cases, ALS patients fell into the categories “insufficient” or “low” levels of vitamin D (Supplementary File 1).

**Table 2:** Description of cohorts retained after the systematic review and included in the meta-analysis.

**Study**	**Number of patients**	**Age (years)**	**Age of onset (years)**	**Men (%)**	**Bulbar form (%)**	**ALSFRS**	**BMI (kg/m^**2**^)**	**Vitamin D levels (ng/ml)**	**Relation vitamin D and ALS**
Gennings et al. (34)	302	63.2	56.2 ± 11.8	59	NR	37	26	NR	Prognosis
Cortese et al. (38)	71	NR	NR	NR	NR	NR	NR	11.0 (4.0–38.0)	Diagnosis Prognosis
Bretón et al. (35)	213	61.9 ± 13.3	BO 67.4 ± 12 SO 59.4 ± 13.2	57	31.5	NR	25.6 ± 4.2	<20	Prognosis (BO x SO)
Trojsi et al. (41)^**^	50.000: 10 75.000: 12 100.000: 11	57.6 (11.69) 62.17 (10.05) 54 (14)	56.5 (11.75) 61.17 (10.49) 53.27 (14.1)	20 58 81	NR	37.6 (6.32) 35.67 (7.83) 38.45 (6.86)	25.35 (2.63) 26.48 (3.29) 23.73 (2.92)	16.7 (6.9) 14.3 (6.5) 14.9 (7.6)	Therapy
Camu et al. (13)	74	66.8^*^	64.3	70	31	0.92 (ASS)^***^	NR	21.2	Prognosis
Karam et al. (33)	37	55	vitD 47.2 (37.3–61) no vitD 60 (47.4–63.3)	57	NR	vitD 29 (16.5–39) no vitD 29 (22–38)	NR	22.3 (13.5–32)	Therapy
Libonati et al. (36)	57	63.0 ± 9.9	NR	82.4	NR	NR	NR	18.8 ± 12.1 ng/dl	Diagnosis Prognosis Therapy
Elf et al. (37)	24	58 ± 12	57.6^*^	58.3	NR	31	25.6 (18.1–43.6)	23.6 ± 10.4	Diagnosis Prognosis
Yang et al. (16)	100	56.3^*^	53.5 (45–63)	59	31	40 (36–43)	22.3 (20.5–24.7)	13.7 ± 8.9	Prognosis
Paganoni et al. (40)	106	58.0 ± 9.8	56.2 ± 9.1^*^	58.5	26.4	33.2 ± 8.4	27.2 ± 5.2	NR	Prognosis
Blasco et al. (15)	125	65 (63–67)	62.4^*^	50.4	34.2	34.4 (33.0–35.7)	24.2 (23.5–24.8)	20.3 (19.8–22.4)	Prognosis
Crick et al. (39)	11	64.5 ± 10.8	NR	63.6	NR	NR	NR	5.2 (6.37)	Diagnosis
Pageot et al. (14)	127	NR	NR	NR	NR	NR	NR	NR	Prognosis
% Data	100	69.2	46.1	84.6	38.5	61.5	53.8	76.9	
Average	98.5	55.7 ± 8.5	57.9 ± 10.7	59.6	30.8	31.4 ± 5.9	25.2 ± 4.3	14.5 ± 8.8	

*Data reported as mean ± S.D, except for Gennings, Karam, Yang, and Blasco who presented data as median*.

*NR, not reported; BO, bulbar onset; SO, spinal onset*.

* *As estimated from the age of the cohort and disease duration or from age at diagnosis and disease duration*.

** *Vitamin D supplementation groups (IU/month)*.

*** *ASS: ALSFRS severity score (mean points lost per month)*.

### Prevalence of Vitamin D Deficiency in Amyotrophic Lateral Sclerosis and Control Individuals

**Figure 2:**
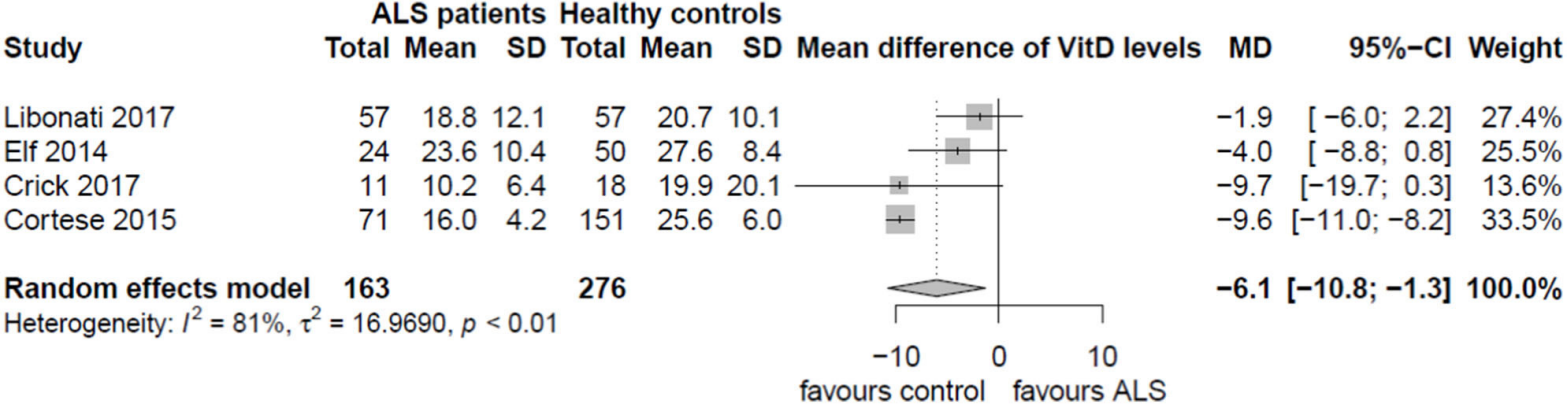
Vitamin D levels in ALS and controls.

Four observational studies reported levels of vitamin D in ALS patients and in control subjects (healthy controls in three studies and other neurological conditions in the fourth) (36–39). Overall, only Cortese et al. (38) reported a significant difference in vitamin D levels between ALS patients and controls. The data available from these four studies (Table 3) were pooled to estimate the mean vitamin D difference between ALS and controls (Figure 2).

**Table 3:** Median (range) of vitamin D levels in ALS and control cases.

**Study**	**Number of patients Type of controls**	**Vit D levels in ALS patients**	**Vit D levels in controls**	***p* value**
Cortese et al. (38)	71 ALS 151 healthy controls	11 ng/ml	22.3 ng/ml (5–52.8)	<0.0001
Libonati et al. (36)	57 ALS 57 healthy controls	18.8 ± 12.1 ng/dl	20.7 ± 10.1 ng/dl	0.369
Elf et al. (37)	24 MND 50 healthy controls	23.64 ± 10.4 ng/ml	27.64 ± 8.41 ng/ml	0.15
Crick et al. (39)	11 ALS 18 controls^*^	10.15 ± 6.37 ng/ml	19.94 ± 20.08 ng/ml	ns

* *Neurological symptoms but no diagnosis at time of sampling*.

*ns, non-significant; MND, motor neuron disease*.

Overall, ALS patients presented lower levels of vitamin D compared with the normal range (>30 ng/ml; 41). In a random-effects model, vitamin D levels in ALS patients were slightly lower than those in control subjects by a mean difference of −6.1 ng/ml; 95% CI (−10.8; −1.3) but with major heterogeneity (*I*^2^ = 81%; *p* < 0.01) introduced mainly by the study of Cortese et al. (38). Heterogeneity could not be explained by any particular characteristic of the study of Cortese and co-workers—apart from its presentation as only an abstract. However, the analysis of bias from retrieved studies raised concerns, as two studies (36, 38) displayed a moderate risk of bias, one study was rated with high risk of bias (39), and only one study (37) presented low risk of bias (Supplementary File 3).

### Vitamin D Levels and Amyotrophic Lateral Sclerosis Prognosis

**Figure 3:**
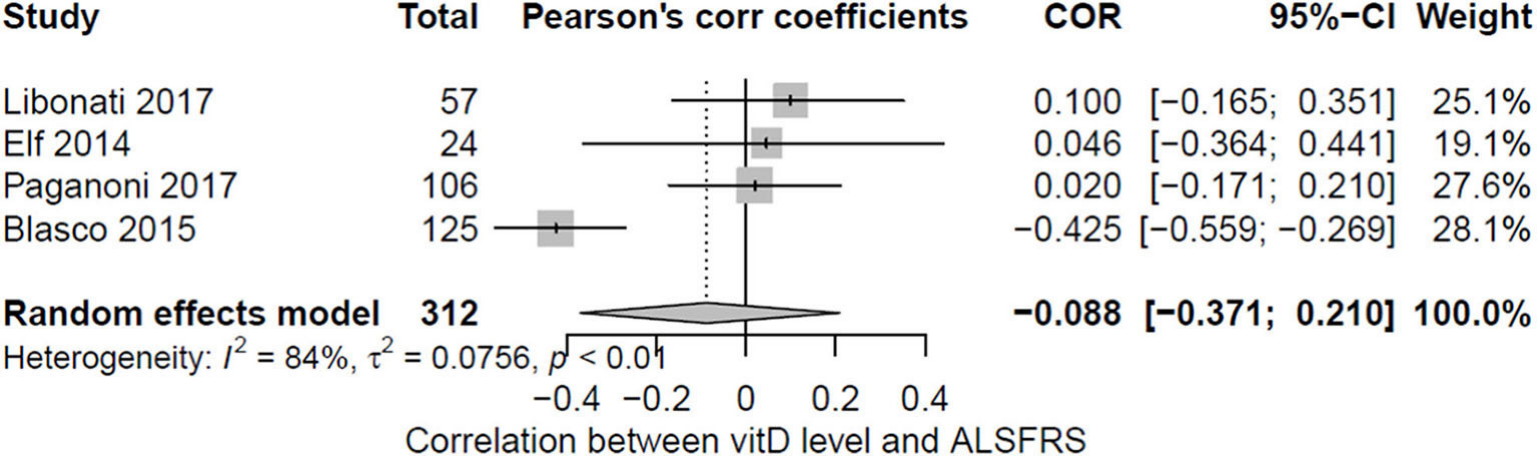
Pooled correlation between vitamin D levels and ALSFRS.

Nine observational studies correlated the levels of vitamin D with a clinical marker used for the evaluation of ALS prognosis (as ALSFRS-R, forced vital capacity, or survival) (13–16, 34, 36–38, 40). Out of these studies, only four (44%) reported sufficient information about the correlation (Pearson's coefficient) between vitamin D levels and ALSFRS and could be pooled (15, 36, 37, 40) (Figure 3). Two other studies reported statistical data about the correlation of ALSFRS and vitamin D levels, but for different reasons, they could not be included in the meta-analysis: Camu et al. (13) reported a correlation between vitamin D levels and a severity score (calculated as the points of ALSFRS-R lost per month), and Cortese et al. performed a Spearman correlation. Interestingly, both studies reported contradictory results: Camu et al. showed a negative correlation, meaning the lower the levels of vitamin D, the higher the severity score; meanwhile, Cortese et al. reported a positive one, meaning the higher the vitamin D levels, the higher the severity score. Overall, no significant correlation between vitamin D and motor dysfunction measured by ALSFRS-R was apparent when pooling the data (−0.088; 95% CI: −0.371; 0.210), with major heterogeneity being found (*I*^2^ = 84%; *p* < 0.01). This discordance in results was also visible when pooling the four studies, with no correlation found in the three studies, whereas one found a significant but negative correlation.

Three studies performed correlations between vitamin D levels and survival, but due to high heterogeneity in reported data, it was not possible to pool them together (Table 4). Studies reported again very conflicting results: whereas Camu et al. reported a positive association between vitamin D levels and survival, Blasco et al. reported a negative effect of vitamin D on survival. On the other hand, Yang reported no significant association between vitamin D levels and survival. The overall quality of studies evaluating the prognostic role of vitamin D (QUIPS tool) was judged to be at high risk of bias, mostly because of the lack of consideration of confounding factors (Table 5). Moreover, three reports of a link between vitamin D levels and prognosis in ALS patients were published as abstracts only (14, 34, 38).

**Table 4:** Correlation of vitamin D levels and survival.

**Study**	**VitD Categories**	***N***	**KM log-rank test**	**Univariate**	Multivariate^*^
Camu et al. (13)	<25 vs. >75 nmol/l	74	*p* = 0.002	Not performed	5.9 (1.4–24.3); *p* = 0.01
Blasco et al. (15)	Per nmol/l Per categories of BMI	125	BMI < median: *P* = 0.014 BMI > median: *P* = 0.022	6.28 (1.88–19.42); *p* = 0.003	BMI < median: 6.6 (1.03–35.8); *p* = 0.047 BMI > median: 6.5 (1.56–28.18); *p*=0.0098
Yang et al. (16)	<10 vs. >10 ng/ml	100	*P* = 0.204	0.63 (0.31–1.31); *p* = 0.221	1.09 (0.466–2.547); *p* = 0.842

*vs., versus; VitD, vitamin D; N, number of patients; KM, Kaplan–Meier*.

* *Multivariate analysis took into account age at onset (13, 16) or ALSFRS-R (15)*.

**Table 5:** Risk of bias assessment using the Quality in Prognosis Studies (QUIPS) tool for studies reporting vitamin D as prognostic in ALS.

**Study**	**Participation**	**Attrition**	**Prognostic factor measurement**	**Outcome measurement**	**Confusion factors**	**Statistical analysis**	**Overall risk of bias**
Elf et al. (37)	Moderate	Low	Moderate	Low	High	High	High
Libonati et al. (36)	High	Low	Moderate	Moderate	High	Moderate	High
Camu et al. (13)	Moderate	Low	Moderate	Moderate	High	Moderate	High
Blasco et al. (15)	Moderate	Low	Moderate	Moderate	Moderate	Moderate	High
Yang et al. (16)	Moderate	Low	Moderate	Low	Moderate	Moderate	High
Paganoni et al. (40)	Low	Low	Moderate	Moderate	Low	Moderate	Moderate
Pageot et al. (14)	Unknown	Unknown	Unknown	Unknown	Unknown	Unknown	Unknown
Gennings et al. (34)	Unknown	Unknown	Unknown	Unknown	Unknown	Unknown	Unknown
Cortese et al. (38)	Unknown	Unknown	Unknown	Unknown	Unknown	Unknown	Unknown

### Vitamin D in Bulbar-Onset and Spinal-Onset Amyotrophic Lateral Sclerosis Patients

Some studies evaluated vitamin D levels in spinal-onset and bulbar-onset ALS. Bretón et al. (35) reported vitamin D deficiency (<20 ng/ml) in 61% of spinal-onset patients (89 from 146) and 37% of bulbar-onset (25 from 67), with no statistical differences between both groups. Libonati et al. (36) also did not find any differences in vitamin D levels between spinal- or bulbar-onset ALS (*p* = 0.9). On the other hand, Cortese et al. (38) reported a statistical difference in vitamin D levels between spinal- and bulbar-onset patients (12.45 vs. 18.50 ng/ml, respectively; *p* = 0.01), and only in spinal-onset patients' vitamin D was positively correlated with ALSFRS (rs = 0.37, *p* = 0.004) and negatively with disease duration (rs = −0.33, *p* = 0.013). Blasco et al. (15) also reported a significant higher level of vitamin D in bulbar-onset than that in spinal-onset ALS patients (spinal: 19.7 [17.6–21.6]; bulbar: 25 [22.2–27.8]; *p* = 0.003).

### Supplementation With Vitamin D and Amyotrophic Lateral Sclerosis Progression

**Figure 4:**
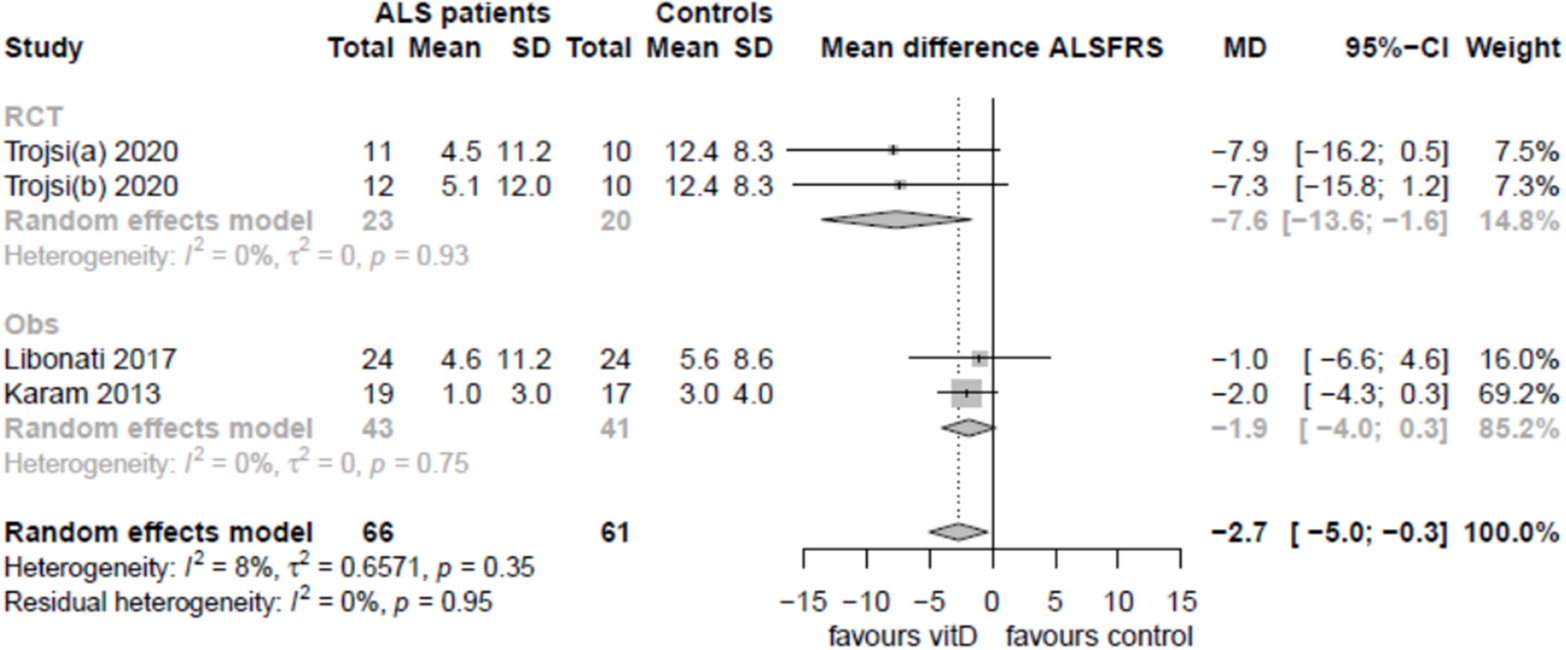
Pooled correlation between vitamin D supplementation and ALSFRS.

Five studies reported information regarding the effect of vitamin D supplementation on ALS progression (15, 33, 36, 40, 41), but only three (60%) reported data that could be pooled: one RCT (41) and two observational, retrospective studies (33, 36) (Figure 4). After 6 months of treatment, no statistical differences were observed regarding the ALSFRS-R progression rate, although serum 25(OH)D levels were increased in treated groups. Whereas, Libonati et al. and Karam et al. compared treated with untreated patients, Trojsi et al. compared groups of patients treated with three different doses of vitamin D. There was no significant effect of vitamin D supplementation in observational studies (mean difference −1.9 [95% CI: −4.0; 0.3]), whereas a higher dose of vitamin D in the only randomized trial slightly improved ALSFRS (mean difference −2.7 [95% CI: −5.0, −0.3]). Analysis of risk of bias for these studies suggests that these results should be considered carefully. A critical risk of bias was found for Libonati et al., serious concerns for Karam et al. (ROBINS-I tool), and some concerns for Trojsi et al. (RoB, 2v tool; Supplementary File 4). An important source of bias in these studies is that ALS patients with vitamin D deficiency were supplemented and then compared with normal vitamin D, non-supplemented ALS patients. Only Trojsi et al. randomized different deficiency levels into all groups of treatment but with non-untreated ALS control, and therefore, results should be considered very carefully.

Importantly, some authors reported deleterious effects of vitamin D supplementation. Paganoni et al. (40) (Caucasian sample, season-averaged vitamin D levels) reported that vitamin D supplementation was associated with a faster decline in the ALSFRS-R by 0.5 points/month (95% CI −0.9 to −0.1 points/month, *p* = 0.018), even after adjusting for covariates. On the other hand, Blasco et al. (15) reported that ALS patients receiving supplementation presented a significantly lower ALSFRS-R (*p* = 0.007).

## Discussion

In this systematic review, we analyzed all studies reporting any relationship between vitamin D and ALS. Thirteen different cohorts totaling 1,280 ALS patients were reported in 12 observational studies and one RCT. We observed a small tendency toward a 6-ng/ml lower vitamin D level in ALS patients compared mainly with that in healthy controls, but data were not adjusted for mean vitamin D level of the general population, and pooled data were highly heterogeneous. The results regarding the relationship between vitamin D levels and ALSFRS-R could be pooled only for 44% of retrieved studies and were highly heterogeneous and inconclusive; similarly, discordant results were reported in three studies regarding survival. Therefore, we do not consider that a vitamin D level could be a biomarker for disease diagnosis or disease severity. Finally, supplementation of ALS patients with vitamin D had no beneficial effect on ALSFRS-R in two observational studies (subject to important confounding either taken into account or residual) and deleterious effects when compared with that in non-treated ALS patients in two other observational studies. Furthermore, one clinical trial reported no statistical benefit of very high doses compared with normal supplementation—but should be very carefully considered because of insufficiency in design and very small sample size.

Heterogeneous results regarding the prognostic and therapeutic role of vitamin D in ALS patients could reflect the diverse potential mechanisms of action of vitamin D in *in vitro*/*in vivo* studies. Its role in preventing ALS progression in *in vitro* studies was demonstrated by different research groups (10, 42). However, in *in vivo* studies, different results were reported. Although vitamin D supplementation seems to improve motor function in a well-known ALS mouse model (SOD1-G93A) (43), and vitamin D deficiency can exacerbate disease pathology (44), it can also promote deleterious effects in motor function and disease severity (45, 46).

Only two studies reported positive effects of vitamin D in ALS patients. Camu et al. reported that patients with severe deficiency of vitamin D have higher severity scores (ALSFRS units lost per month), and patients with normal levels of vitamin D have increased survival. Karam et al. investigated the possible effects of vitamin D supplementation, and although no beneficial effect was reported at month 6 after starting supplementation, they found a positive effect of vitamin D on ALSFRS only after 9 months of supplementation. From the three retained studies, which investigated the effect of vitamin D supplementation in improving motor symptoms, the other two (36, 41) established the final outcome at month 6.

Several important points need to be considered when analyzing vitamin D levels in patients with neurodegenerative diseases, as these patients may suffer from low vitamin D because they lack sunlight exposure and physical activity (especially the ones with more restricted movement, as it is the case for ALS patients) (41). In the study of Camu et al. (13), however, the authors excluded patients who could not walk without assistance, as this handicap could induce a lower sun exposure and, thus, lower vitamin D levels. Interestingly, this study found a positive correlation between vitamin D and survival, whereas low vitamin D was correlated with faster disease progression measured by the ALSFRS severity score (13). However, the Camu et al. study is not representative of all French ALS patients, as another study from Blasco et al. (15) showed no protective effect of vitamin D in another cohort of French ALS patients.

In a Swedish cohort (37), the motor neuron disease group presented a tendency of higher vitamin D levels in the summer, although not significant (*p* = 0.057). This study included seasoned-averaged vitamin D levels, as did Paganoni et al. (40)—both studies did not find positive correlations between vitamin D levels and ALSFRS-R score. Variations in the levels of vitamin D regarding different seasons should be considered, especially in populations where daytime sunlight changes substantially between seasons. Another consideration is that the vitamin D levels considered as “normal” can change from country to country and in the function of ethnicity. This variability is taken into account in the study of Yang et al. (16), who reported the mean of vitamin D levels for the Korean population as being 19.6 ± 6.9 ng/ml (47)—a number considered low in comparison with that in other countries and ethnicities (48). Furthermore, a systematic review reported the lack of available worldwide data for vitamin D levels in the different populations, and when data were available, they report vitamin D deficiency as a global public health problem in all age groups (49).

Other important factors of variability in the levels of vitamin D, such as bone density, calcium metabolism, and kidney function, were not considered in the majority of analyzed studies. Renal function was assessed in the study of Cortese et al. who stated that in both control and ALS patients, kidney function was normal. In addition, Yang et al. investigated bone mineral density and calcium metabolism in ALS patients, and although they reported very low levels of vitamin D in the ALS cohort analyzed, vitamin D was not a predictor of survival.

As low levels of vitamin D seems to be a global public health problem regardless of age, vitamin D supplementation should be combined with increased sun exposure with the purpose of increasing overall health and also to avoid bone fractures in the elderly but not with the aim to treat the symptoms of neurodegenerative diseases.

## Conclusion

Our analyses suggest that there is a tendency for ALS patients to have lower levels of vitamin D than controls, but most of the studies did not adjust for the mean vitamin D of the country population and failed to report important factors of variability for vitamin D levels (for example, lack of sun exposure). When studies presented enough data to be analyzed, our meta-analyses did not find strong evidence for a relationship between vitamin D levels and prognosis, nor did vitamin D supplementation induce an improvement in motor function. Our study does not support any particular positive link between vitamin D and ALS. Furthermore, the risk of bias analysis raised some concerns about the quality of these studies. ALS patients should receive vitamin D supplementation for other health issues related to vitamin D deficiency, but there is not enough evidence to support vitamin D as a therapy for ALS.

## Data Availability Statement

The datasets generated for this study are available on request to the corresponding author.

## Author Contributions

DL, TB-A, and HB contributed to the conception and design of the study. DL and JG organized the database. DL and TB-A performed the statistical analysis. DL wrote the first draft of the manuscript. TB-A, JG, HB, and PC wrote sections of the manuscript. All authors contributed to manuscript revision, read, and approved the submitted version.

## Conflict of Interest

The authors declare that the research was conducted in the absence of any commercial or financial relationships that could be construed as a potential conflict of interest.
