# Supplementary material for: Is There a Role for Vitamin D in Amyotrophic Lateral Sclerosis? A Systematic Review and Meta-Analysis
Source: Front Neurol. 2020 Jul 31;11:697. doi: 10.3389/fneur.2020.00697 (PMC7411408; doi:10.3389/fneur.2020.00697)
Supplement: Supplementary file 1 [file Table_1.docx]

**Supplementary Table 1**. Distribution of ALS patients (%) in regard of vitamin D categories (insufficient: <20 ng/mL; low: 20-30 ng/mL; normal: >30 ng/mL).

| **Study** | | **Insufficient** | **Low** | **Normal** |
| --- | --- | --- | --- | --- |
| Libonati et al. (2017) | ALS | 59.6 | 31 | 8.8 |
| Trojsi et al. (2020) | ALS | 67 | 33 | - |
| Paganoni et al. (2017) | ALS | 19 | 50 | 30 |
| Karam et al. (2013) | ALS | 43 | 38 | 19 |
| Camu et al. (2014) | ALS | 14 | 70 | 16 |
| Elf et al. (2014) | MND | 4 | 75 | 21 |
| Yang et al. (2016) | ALS | 34 | 66 | - |

*<25 nmol/L, >25 and <75, >75 nmol/L; # insufficient: <10 ng/mL, low: > 10 ng/mL.

**References**

Camu, W., Tremblier, B., Plassot, C., Alphandery, S., Salsac, C., Pageot, N., et al. (2014). Vitamin D confers protection to motoneurons and is a prognostic factor of amyotrophic lateral sclerosis. *Neurobiol Aging* 35(5)**,** 1198-1205. doi: 10.1016/j.neurobiolaging.2013.11.005.

Elf, K., Askmark, H., Nygren, I., and Punga, A.R. (2014). Vitamin D deficiency in patients with primary immune-mediated peripheral neuropathies. *J Neurol Sci* 345(1-2)**,** 184-188. doi: 10.1016/j.jns.2014.07.040.

Karam, C., Barrett, M.J., Imperato, T., MacGowan, D.J., and Scelsa, S. (2013). Vitamin D deficiency and its supplementation in patients with amyotrophic lateral sclerosis. *J Clin Neurosci* 20(11)**,** 1550-1553. doi: 10.1016/j.jocn.2013.01.011.

Libonati, L., Onesti, E., Gori, M.C., Ceccanti, M., Cambieri, C., Fabbri, A., et al. (2017). Vitamin D in amyotrophic lateral sclerosis. *Funct Neurol* 32(1)**,** 35-40. doi: 10.11138/fneur/2017.32.1.035.

Paganoni, S., Macklin, E.A., Karam, C., Yu, H., Gonterman, F., Fetterman, K.A., et al. (2017). Vitamin D levels are associated with gross motor function in amyotrophic lateral sclerosis. *Muscle Nerve* 56(4)**,** 726-731. doi: 10.1002/mus.25555.

Trojsi, F., Siciliano, M., Passaniti, C., Bisecco, A., Russo, A., Lavorgna, L., et al. (2020). Vitamin D supplementation has no effects on progression of motor dysfunction in amyotrophic lateral sclerosis (ALS). *Eur J Clin Nutr* 74(1)**,** 167-175. doi: 10.1038/s41430-019-0448-3.

Yang, J., Park, J.S., Oh, K.W., Oh, S.I., Park, H.M., and Kim, S.H. (2016). Vitamin D levels are not predictors of survival in a clinic population of patients with ALS. *J Neurol Sci* 367**,** 83-88. doi: 10.1016/j.jns.2016.05.007.
