# Supplementary material for: Is There a Role for Vitamin D in Amyotrophic Lateral Sclerosis? A Systematic Review and Meta-Analysis
Source: Front Neurol. 2020 Jul 31;11:697. doi: 10.3389/fneur.2020.00697 (PMC7411408; doi:10.3389/fneur.2020.00697)
Supplement: Supplementary file 3 [file Data_Sheet_2.docx]

**Risk of bias tool – Hoy et al. 2012**

This tool is designed to assess the risk of bias in population-based prevalence studies.

Note: If there is insufficient information in the article to permit a judgement for a particular item, please answer **No (HIGH RISK)** for that particular item.

**Study:** Cortese et al. 2015. Vitamin D levels in serum of ALS patients (P2.069)

| **Risk of bias item** | **Criteria for answers** | **Additional notes** |
| --- | --- | --- |
| *External validity* | | |
| 1. was the study’s target population a close representation of the national  population in relation to relevant variables, e.g. age, sex, occupation? | No; high risk | Characteristics of participants or where the study was conducted was not informed in the abstract. |
| 2. Was the sampling frame a true or close representation of the target population? | No; high risk | Characteristics of participants or where the study was conducted was not informed in the abstract. |
| 3. Was some form of random selection used to select the sample, OR, was a census undertaken? | No; high risk | Characteristics of participants or where the study was conducted was not informed in the abstract. |
| 4. Was the likelihood of non-response bias minimal? | Yes; low risk | No information about non-response |
| 5. Were data collected directly from the subjects (as opposed to a proxy)? | Yes; low risk |  |
| 6. Was an acceptable case definition used in the study? | Yes; low risk | ALS patients – Escorial criteria |
| 7. Was the study instrument that measured the parameter of interest shown to have reliability and validity (if necessary)? | Yes; low risk | Vit D: CLIA; ALS: ALSFRS, disease duration. |
| 8. Was the same mode of data collection used for all subjects? | Yes; low risk | Probably |
| 9. Was the length of the shortest prevalence period for the parameter of interest appropriate? | No; high risk | Not informed |
| 10. Were the numerator(s) and  denominator(s) for the parameter of interest appropriate? | Yes; low risk | Healthy controls vs ALS patients |
| ***11. Summary item on the overall risk of study bias:* moderate** | | |

**Study:** Elf et al. 2014. Vitamin D deficiency in patients with primary immune-mediated peripheral neuropathies (J. Neurolog Sci)

| **Risk of bias item** | **Criteria for answers** | **Additional notes** |
| --- | --- | --- |
| *External validity* | | |
| 1. was the study’s target population a close representation of the national  population in relation to relevant variables, e.g. age, sex, occupation? | No; high risk | The study was conducted in one hospital only, and it is not clear if this was representative of the national population. |
| 2. Was the sampling frame a true or close representation of the target population? | Yes; low risk | Not clear, but possibly yes |
| 3. Was some form of random selection used to select the sample, OR, was a census undertaken? | Yes; low risk | All patients followed in the clinic were included in the study. |
| 4. Was the likelihood of non-response bias minimal? | Yes; low risk | No information about non-response |
| 5. Were data collected directly from the subjects (as opposed to a proxy)? | Yes; low risk |  |
| 6. Was an acceptable case definition used in the study? | Yes; low risk | MND patients with planned visits |
| 7. Was the study instrument that measured the parameter of interest shown to have reliability and validity (if necessary)? | Yes; low risk | Vit D: CLIA; ALS: ALSFRS. |
| 8. Was the same mode of data collection used for all subjects? | Yes; low risk | Probably |
| 9. Was the length of the shortest prevalence period for the parameter of interest appropriate? | No; high risk | Not informed |
| 10. Were the numerator(s) and  denominator(s) for the parameter of interest appropriate? | Yes; low risk | Healthy controls vs ALS patients |
| ***11. Summary item on the overall risk of study bias:* low** | | |

**Study:** Libonati et al. 2017. Vitamin D in ALS (Functional Neurol)

| **Risk of bias item** | **Criteria for answers** | **Additional notes** |
| --- | --- | --- |
| *External validity* | | |
| 1. was the study’s target population a close representation of the national  population in relation to relevant variables, e.g. age, sex, occupation? | No; high risk | The study was conducted in one hospital only, and it is not clear if this was representative of the national population. |
| 2. Was the sampling frame a true or close representation of the target population? | No; high risk | Not clear, but possibly yes |
| 3. Was some form of random selection used to select the sample, OR, was a census undertaken? | No; high risk | Retrospective analysis of patients with low and normal vitD levels |
| 4. Was the likelihood of non-response bias minimal? | Yes; low risk | No information about non-response |
| 5. Were data collected directly from the subjects (as opposed to a proxy)? | Yes; low risk | Collected from medical folders |
| 6. Was an acceptable case definition used in the study? | Yes; low risk | Definite or probable ALS |
| 7. Was the study instrument that measured the parameter of interest shown to have reliability and validity (if necessary)? | Yes; low risk | Vit D: CLIA; ALS: ALSFRS. |
| 8. Was the same mode of data collection used for all subjects? | Yes; low risk | Probably |
| 9. Was the length of the shortest prevalence period for the parameter of interest appropriate? | No; high risk | Not informed |
| 10. Were the numerator(s) and  denominator(s) for the parameter of interest appropriate? | Yes; low risk | Healthy controls vs ALS patients |
| ***11. Summary item on the overall risk of study bias:* moderate** | | |

**Study:** Crick et al. 2017. Reduced plasma levels of 25-hydroxycholesterol and increased CSF levels of bile acid precursors in multiple sclerosis patients (Mol Neurobiol).

| **Risk of bias item** | **Criteria for answers** | **Additional notes** |
| --- | --- | --- |
| *External validity* | | |
| 1. was the study’s target population a close representation of the national  population in relation to relevant variables, e.g. age, sex, occupation? | No; high risk | The study was conducted in one hospital only, and it is not clear if this was representative of the national population. |
| 2. Was the sampling frame a true or close representation of the target population? | No; high risk | Not informed |
| 3. Was some form of random selection used to select the sample, OR, was a census undertaken? | No; high risk | Not informed |
| 4. Was the likelihood of non-response bias minimal? | Yes; low risk | No information about non-response |
| 5. Were data collected directly from the subjects (as opposed to a proxy)? | Yes; low risk |  |
| 6. Was an acceptable case definition used in the study? | No; high risk | Not informed |
| 7. Was the study instrument that measured the parameter of interest shown to have reliability and validity (if necessary)? | Yes; low risk | Vit D: CLIA; ALS: not informed |
| 8. Was the same mode of data collection used for all subjects? | Yes; low risk | Probably |
| 9. Was the length of the shortest prevalence period for the parameter of interest appropriate? | No; high risk | Not informed |
| 10. Were the numerator(s) and  denominator(s) for the parameter of interest appropriate? | No; high risk | Controls were neurological patients with no diagnosis |
| ***11. Summary item on the overall risk of study bias:* high** | | |
